# Dr. Lebert on the Structure of Cataract

**Published:** 1852-03

**Authors:** 


*Dr. Lebert on the Structure of Cataract.*—Dr. Lebert stated, a short time ago, before the Surgical Society of Paris, that he had no faith in certain ammoniacal preparations which had been supposed capable of producing resolution in case of incipient cataract. He examined crystalline lenses somewhat altered in structure, and found very important changes. In hard cataract, for instance, there was an opaque, granular substance interposed between the lamellæ of the lens; this substance is beyond the action of the absorbents, as the lamellæ themselves are horny and atrophied. In soft cataract there is seen in the crystalline cells an effusion of a milky fluid, and in this fluid crystals of cholestearine can be distinguished, the lamellæ being at the same time softened and hypertrophied. None but a surgical treatment can, in such cases, be followed by a successful result. It was shown during the discussion that those cases which were benefitted by Gondret's ammonical ointment, were not cases of incipient cataract, but instances of an early stage of amaurosis.—*Ibid.*

